# Prevalence and drug resistance patterns of Gram-negative enteric bacterial pathogens from diarrheic patients in Ethiopia: A systematic review and meta-analysis

**DOI:** 10.1371/journal.pone.0265271

**Published:** 2022-03-16

**Authors:** Achenef Melaku Beyene, Mucheye Gezachew, Desalegn Mengesha, Ahmed Yousef, Baye Gelaw

**Affiliations:** 1 Department of Medical Microbiology, College of Medicine and Health Sciences, University of Gondar, Gondar, Ethiopia; 2 Global One Health Initiative, East African Regional Office, Addis Ababa, Ethiopia; 3 Department of Food Science and Technology, Ohio State University, Ohio, Columbus, United States of America; University of Lincoln, UNITED KINGDOM

## Abstract

**Background:**

Diarrhoea is the leading cause of morbidity and mortality in the world particularly in developing countries and among vulnerable groups of the population. Gram-negative enteric bacterial pathogens (GNEBPs) are a group of organisms that reside mainly in the intestine and induce diarrhoea. Antimicrobial agents are usually the part of their treatment regimen. The therapeutic effect of antimicrobials is hindered by the emergence and spread of drug-resistant strains. The information regarding the prevalence and antimicrobial resistance patterns of GNEBPs in Ethiopia is limited and found in a scattered form.

**Objectives:**

This study was designed to determine the pooled prevalence and drug resistance patterns of GNEBPs by meta-analysis of data from diarrhoeic patients in Ethiopia.

**Method:**

A comprehensive literature search was conducted through internet searches using Google Scholar, PubMed, Science Direct, HINARI databases, and reference lists of previous studies. Published articles were included in the study based on priorly set inclusion and exclusion criteria. Results were presented in the forest plot, tables, and figures with a 95% confidence interval (CI). The inconsistency index (I^2^) test statistics was used to assess heterogeneity across studies. The pooled prevalence estimate of GNEBPs and their drug resistance patterns were computed by a random-effects model. Software for Statistics and Data Science (STATA) version 14 statistical software was used for the analysis.

**Result:**

After removing those articles which did not fulfil the inclusion criteria, 43 studies were included in the analysis. Studies were conducted in 8 regions of the country and most of the published articles were from the Amhara region (30.23%) followed by Oromia (18.60%) and Southern Nations, Nationalities, and Peoples’ region (SNNP) (18.60%). The pooled prevalence of GNEBPs was 15.81% (CI = 13.33–18.29). The funnel plot indicated the presence of publication bias. The pooled prevalence of GNEBPs in Addis Ababa, Amhara, SNNP, and Oromia regions were 20.08, 16.67, 12.12, and 11.61%, respectively. The pooled prevalence was 14.91, 18.03, and 13.46% among studies conducted from 2006–2010, 2011–2015, and 2016–2021, respectively and it was the highest (20.35%) in children having age less than or equal to 15 years. The pooled prevalence of *Escherichia coli*, *Campylobacter* spp., *Shigella* spp., and *Salmonella enterica* were 19.79, 10.76, 6.24, and 5.06%, respectively. Large proportions (60–90%) of the isolates were resistant to ampicillin, amoxicillin, tetracycline, and trimethoprim-sulphamethoxazole. The pooled prevalence of multidrug resistance (MDR) was 70.56% (CI = 64.56–76.77%) and MDR in *Campylobacter* spp., *Shigella* spp., *E*. *coli*, and *S*. *enterica*. were 80.78, 79.08, 78.20, and 59.46%, respectively.

**Conclusion:**

The pooled estimate showed a high burden of GNEBPs infections and a high proportion of drug resistance characters to commonly used antimicrobial agents in Ethiopia. Therefore, performing drug susceptibility tests, establishing an antimicrobial surveillance system and confirmation by molecular techniques are needed.

## Introduction

World Health Organization (WHO) defines diarrhoea as the passage of three or more loose or liquid stools per day (24 hours). During diarrhoea, the water content and volume of stool and defecation frequency will usually increase. The syndrome may be accompanied by other illnesses like vomiting, fever, dysentery, nausea, and abdominal cramps. It is the leading cause of morbidity and mortality in the world and contributes about 4% of all deaths and 5% of health loss to disability [1–3]. Diarrhoea is the fifth leading cause of death and it contributes to one in nine deaths among children younger than 5 years [4,5]. The problem is severe among the vulnerable population such as children, people with HIV, the elderly, and other individuals having weak immunity. Many factors contribute to diarrhoea; however, childhood wasting (low weight-for-height score), unsafe water, and unsafe sanitation are the leading risk factors [5]. The incidence of diarrhoea is different among the regions or continents of the world. It is highly prevalent in Sub-Saharan Africa and South Asia. The report from WHO showed that these countries account for about 78% of all diarrheal deaths among children in the developing world [2,4]. Ethiopia is one of the top three countries with very high child mortality due to diarrhoea in Africa [5–7].

Diarrhoea can be induced by a variety of causes. However, infectious agents like viruses and bacteria are among the leading causes. Bacteria, particularly Gram-negative enteric bacterial pathogens (GNEBPs) are the common causes of the syndrome. The group includes bacteria that reside mainly in the intestine. Genera such as *Escherichia*, *Shigella*, *Campylobacter*, *Salmonella*, *Enterobacter*, *Klebsiella*, *Yersinia*, *Serratia*, *Proteus*, and others are included in the group. However, the most common and significant pathogens are *S*. *enterica*, *E*. *coli*, *Campylobacter*, and *Shigella* spp. [8].

Antimicrobial agents are usually part of the treatment regimen, particularly on diarrhoea caused by bacteria. Due to the widespread and indiscriminate use of antimicrobials, several resistant strains are emerging which tend to spread globally [9,10]. Hence, antimicrobial resistance (AMR) is a global health threat and was recognized in the 2016 United Nations (UN) General Assembly [11]. It is one of the top challenges in achieving the 2030 UN sustainable development goals [10]. Infections caused by resistant organisms affect treatment outcomes, treatment costs, disease spread, and duration of illness, posing a challenge to the future of chemotherapy [12]. Some pathogenic strains are also developing resistance not only to one but to several agents, i.e., multidrug resistance [13,14].

Information regarding the prevalence and antimicrobial resistance patterns of GNEBPs in Ethiopia is limited, and available information is found in scattered forms. Hence, there is an interest to conduct a nationwide study. To fill this significant gap, this systemic review and meta-analysis was prepared. The review focused on the prevalence and antimicrobial resistance patterns of GNEBPs isolated from diarrheic patients in Ethiopia. The output of this systematic review and meta-analysis can be used by clinicians, policymakers, and researchers to make evidence-based decisions.

## Methods

### Literature search and selection

The published articles were searched based on preferred reporting items for systematic reviews and meta-analyses (PRISMA) guideline [15]. The search was performed from June to August 2021 using Google Scholar, PubMed, Science Direct, and HINARI databases. The search queries were set based on medical subject headlines (MESH) and Boolean logic. Relevant MeSH terms and keywords were used to retrieve all relevant articles from the databases listed above. The keywords and MeSH terms used were “enteric bacteria AND diarrhoea AND drug resistance AND Ethiopia”, “*Salmonella* AND diarrhoea, AND Ethiopia AND drug resistance”, “*Shigella* AND diarrhoea AND Ethiopia AND drug resistance”, “*Escherichia coli* AND diarrhoea AND Ethiopia AND drug resistance”, “*Campylobacter* AND diarrhoea AND Ethiopia AND drug resistance” “*Yersinia* AND diarrhoea AND Ethiopia AND drug resistance”. Each bacterial genus was searched separately, and a search was also conducted on reference lists of previous studies to increase the chance of getting more articles. Only those articles which fulfil the selection criteria were used to analyse the information.

### Inclusion and exclusion criteria

Research conducted on GNEBPs from the diarrhoeic patient or their antimicrobial susceptibility in Ethiopia and full-length published articles in the English language were included in the analysis. To get updated information on the issue, articles published from 2010 to August 2021 were considered. Studies that did not focus on GNEBPs from the diarrhoeic patient or their antimicrobial susceptibility, anonymous reports, abstracts (incomplete information), and published articles before 2010 and unpublished information were not included in the study. Studies that were conducted to assess the knowledge, attitude, and practice (KAP) of the community or the professionals were not also included.

### Data extraction

The selected articles were coded, and data were collected using a format prepared in Microsoft Excel. The format consists of the author’s name, study period, year of publication, study design, study region, study population, sample size, sample type, age, gender, isolated bacteria species, their prevalence, resistance patterns of the isolates, and prevalence of multidrug resistance. The extracted data were checked at least twice for their accuracy.

### Quality control

The quality of eligible studies was checked using a set of criteria based on Joanna Briggs Institute critical appraisal tools including appropriateness of the research design to address the target population, adequate sample size, quality of paper, completeness of the information, and appropriateness of methods for isolation of the bacteria and appropriate statistical analysis [16]. The eligibility of selected articles was also assessed and approved by experts in the discipline.

### Data analysis

The data were compiled in Excel 2010 (Microsoft, Redmond, WA, USA) spreadsheet and summarized by descriptive statistics. A random-effect model was used to determine the pooled prevalence and the 95% confidence interval (CI). All statistical analysis was achieved by using Software for Statistics and Data Science (STATA; https://www.stata.com/company/our-sites/) version 14. The data were described using forest plots, figures, and tables. The presence of publication bias was assessed by funnel plot. Sub-group analysis was performed based on the regions in the country, age group; (children, adults, and all age groups), and year of study (2006–2010, 2011–2015, and 2016–2021). Statistical heterogeneity was evaluated by the inconsistency index (I^2^) test. The I^2^ provides an estimate of the percentage of the variability in effect estimates that is due to heterogeneity rather than sampling error or chance differences. Hence, the I^2^ test measures the level of statistical heterogeneity among studies [17,18].

## Results

### Characteristics of published articles

**Fig 1:**
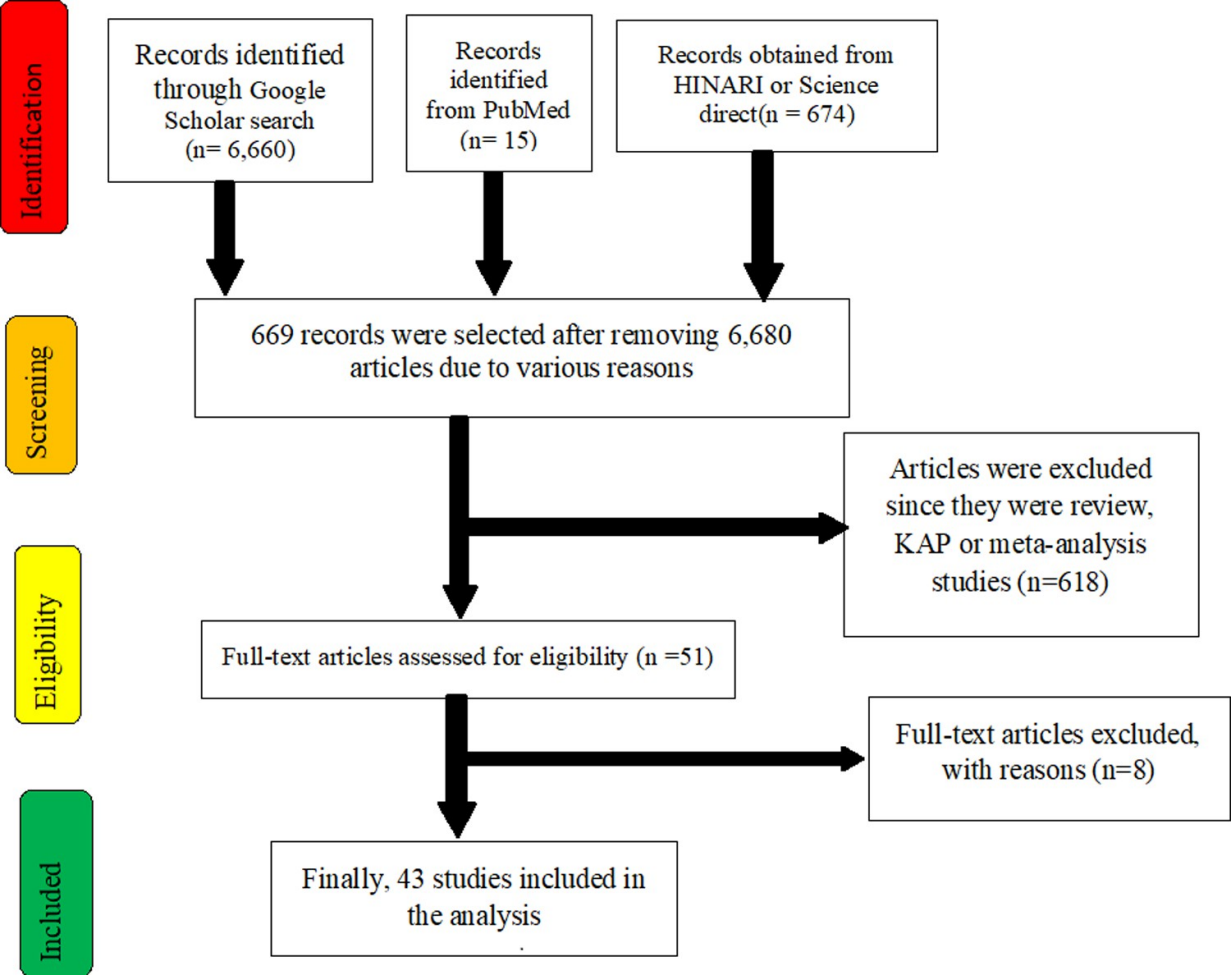
A flow diagram that shows the selection of articles for the analysis.

Of 7,349 identified studies, 6,680 articles were excluded upon reviewing the titles and abstracts because they were irrelevant (were not focusing on GNEBPs, diarrhoea, and drug resistance or were outside Ethiopia or duplicates). The remaining 669 articles were assessed for eligibility; of these, 626 articles were excluded since they were review, KAP, or meta-analysis studies. Finally, 43 studies meeting the inclusion criteria were included in this study. Selected articles were focusing on one or more GNEBPs. Fig 1 shows a flow diagram of the selection of articles for the analysis.

**Fig 2:**
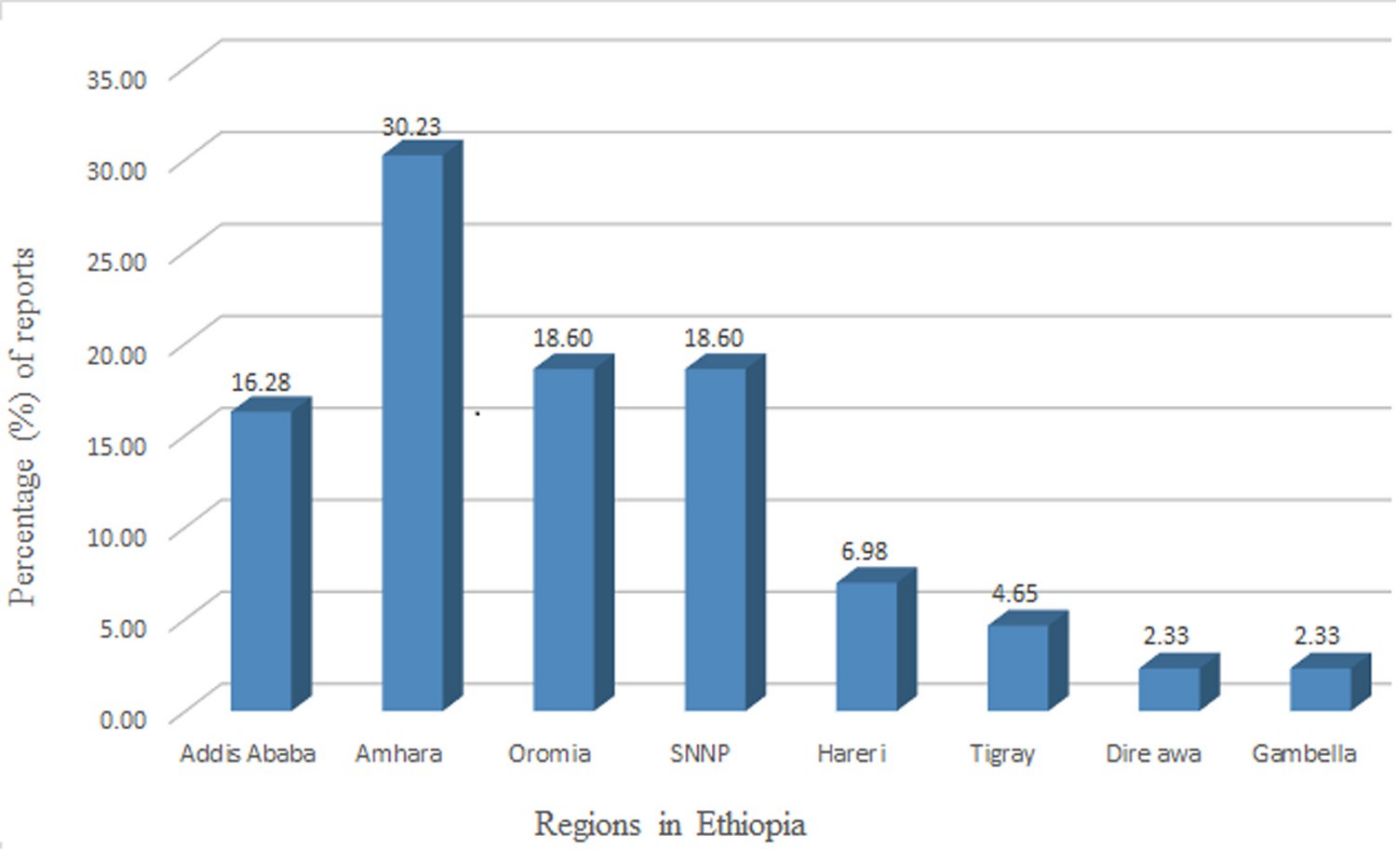
Percent of published articles in different regions of Ethiopia. The country has 9 regions and two city administrations. Reports were from 8 of them which are indicated in the figure. Reports were not found from Afar, Somali, and Benishangul Gumuz regions during the period of data collection (SNNP = South Nation and Nationalities Region).

Table 1 shows the overall characteristics of articles included in the analysis, type and prevalence of Gram-negative isolates recovered from diarrheic patients. Studies were conducted in 8 regions of the country and most of the published articles were from the Amhara region (30.23%) followed by the Oromia region (18.60%) and South Nation and Nationalities Region (SNNP) (18.60%). Published articles were not found in other regions of the country (Afar, Somali, and Benishangul Gumuz regions) (Fig 2).

**Table 1:** Characteristics, quality, and the number of Gram-negative isolates recovered from diarrheic patients.

References	Year of publication	Study period	Region	Study population	Age category	Gender		Number examined	No Positive	Prevalence	*E*. *coli*	*Salmonella*	*Shigella*	*Klebsiella*	*Proteus*	*Enterobacter*	*Campylobacter*	*Citrobacter*
						Male	Female											
[19]	2018	Aug–Dec 2015	Addis Ababa	Diarrheic patient	<15 yrs	115	138	253	94	37.15	61	23	10					
[20]	2021	Mar 2019 to Nov 2019	SNNP	diarrheic patient	Adult > 15yrs	151	127	278	24	8.63		15	9					
[21]	2018	Nov 2015 and Aug 2016	Amhara	diarrheic patient	<15 yrs	99	64	163	91	55.83	47	5	3	11	7	2		
[22]	2020	Jan to March 2018	Amhara	Diarrheic patient, HIV +	all	163	191	354	24	6.78		17	7					
[23]	2020	Jan to July 2014	Oromia	diarrheic children	<15 yrs	125	114	239	9	3.77		3	6					
[24]	2018	June to Sept 2017	SNNP	diarrheic patient	<15 yrs	101	103	204	19	9.31		3	17					
[25]	2011	Aug to Nov 2009	Amhara	diarrheic patient	all	125	90	215	32	14.88			32					
[26]	2014	Feb to May, 2014	Amhara	diarrheic patient	all	180	192	372	21	5.65		4	17					
[27]	2018	June to Oct, 2016	Amhara	diarrheic patient	<15 yrs	68	44	112	4	3.57		1	3					
[28]	2014	March to Nov 2012	Oromia	diarrheic patient	<15 yrs	114	146	260	22	8.46		16	6					
[29]	2015	March to May 2011	Oromia	diarrheic patient	<15 yrs	14	10	24	7	29.17			7					
[30]	2020	March and Aug 2019	SNNP	HIV infected diarrheic	Adult >15 yrs	84	96	180	15	8.33		5	2				8	
[13]	2011	Jan to Aug 2006	Addis Ababa	diarrheic patient	<15 yrs	654	571	1225	126	10.29		65	61					
[31]	2019	Nov 2016 and May 2017	Addis Ababa	diarrheic patient	<15 yrs	155	135	290	42	14.48	13	7	22					
[32]	2013	Oct 2011 to March 2012	Amhara	diarrheic patient	<15 yrs	144	141	285	44	15.44							44	
[33]	2015	Dec 2011 to Feb 2012	Amhara	diarrheic patient	<15 yrs	239	183	422	73	17.30		33	40					
[34]	2014	Feb to May 2011	Harari	diarrheic patient	all	193	191	384	56	14.58			56					
[35]	2014	June to Oct, 2011	SNNP	diarrheic patient	<15 yrs	81	77	158	35	22.15		4	11				20	
[36]	2014	Dec 2011 to Feb 2012	Amhara	diarrheic patient	<15 yrs			422	33	7.82		33						
[37]	2019	Feb 2017 to March 2017	Oromia	diarrheic patient	all	99	133	232	42	18.10		22	20					
[38]	2020	Nov 2016 to Jan 2017	Amhara	diarrheic patient	all	181	203	384	20	5.21		20						
[39]	2015	Dec 2011 to Feb 2012	Amhara	diarrheic patient	<15 yrs	183	239	422	204	48.34	204							
[40]	2016	Dec 2013 to Mar 2014	Addis Ababa	diarrheic patient	<15 yrs	125	131	256	78	30.47	78	7	33					
[41]	2019	January to March 2018	Amhara	diarrheic patient	<15 yrs	151	121	272	29	10.66			29					
[42]	2018	Oct 2015 to Feb 2016	Oromia	diarrheic patient	all	223	199	422	39	9.24		30	9					
[43]	2014	July to Oct 2012	Oromia	diarrheic patient	<15 yrs	106	121	227	38	16.74							38	
[44]	2018	Mar to May, 2017	SNNP	diarrheic patient	<15 yrs	95	72	167	29	17.37		21	8					
[45]	2019	June to Dec 2017	Gambella	diarrheic patient	<15 yrs	74	60	134	55	41.04		4	14					
[46]	2015	August to Nov 2014	Tigray	diarrheic patient	all	109	107	216	15	6.94			15					
[47]	2014	Oct 2011 to June 2012	SNNP	diarrheic patient	all	221	161	382	57	14.92		40	17					
[48]	2011	Jan to Feb 2007	Harari	diarrheic patient	all	119	125	244	45	18.44		28	17					
[49]	2019	April to July 2016	Oromia	diarrheic patient	<15 yrs	179	243	422	47	11.14		29	18					
[50]	2018	Nov 2011 to March 2012	Tigray	diarrheic patient	<15 yrs	145	115	260	37	14.23		19	18					
[51]	2011	Oct 2006 to March 2007	Amhara	diarrheic patient	All	180	204	384	66	17.19		60	6					
[52]	2021	Apr to Aug 2019	SNNPR	diarrheic patient	All	130	133	263	21	7.98		1	20					
[53]	2015	May 2013 to Jan 2014	Addis Ababa	Diarrheic patient	All	425	532	957	59	6.17		59						
[54]	2020	January 2017	Amhara	Diarrheic patient	<15 yrs	152	192	344	45	13.08	35	6	4					
[55]	2016	-	Oromia	Diarrheic patient	all	84	92	176	21	11.93		19	2					
[56]	2015	Aug.-Dec. 2012	Addis Ababa	diarrheic patient	<15 yrs	115	138	253	104	41.11	61	10	23					10
[57]	2017	Feb to May, 2016	SNNP	HIV-infected with Gastroenteritis	all	103	112	215	27	12.56	2	11	3				13	
[58]	2018	Dec. 2014 to March 2015	Dire Dawa	Diarrheic patient	<15 yrs	105	91	196	43	21.94	25	7	11					
[59]	2021	-	Addis Ababa	diarrheic patient	All	176	122	298	14	4.70		14						
[60]	2014	-	Harari	diarrheic patient	All	208	176	384	56	14.58		56						

yrs = years, SNNP = Southern Nations, Nationalities, and Peoples’ Region.

**Fig 3:**
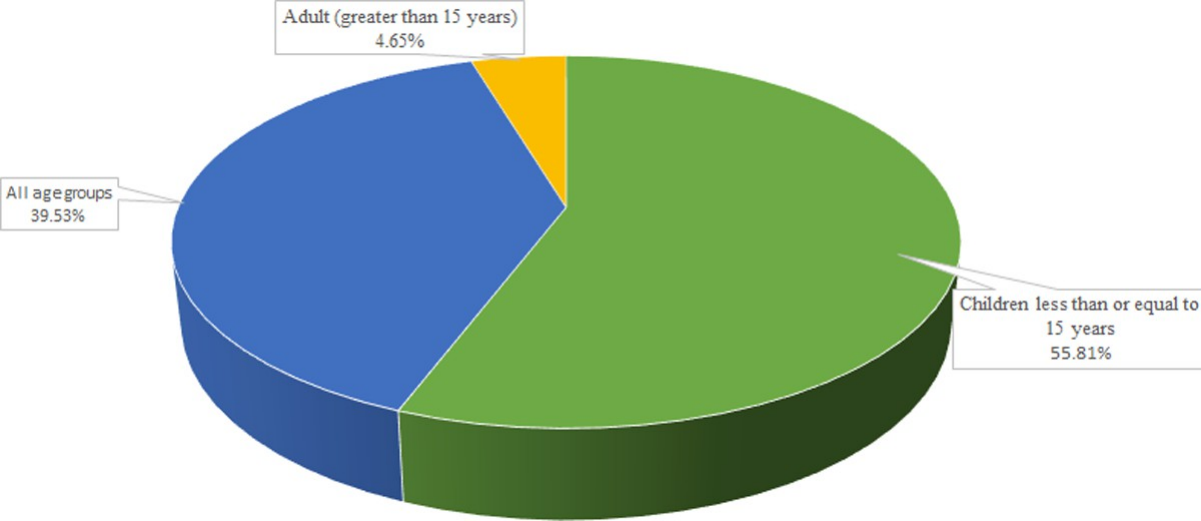
Age categories and percentage of published articles.

All studies were cross-sectional; conducted from 2006 to 2020 and published online from 2010 to August 2021. Almost all studies (97.67%) were institution-based (conducted on patients visiting health facilities). The stool samples were the specimen used to isolate the bacterial species. Isolates were characterized and their identities were confirmed by cultural and conventional biochemical tests, but molecular techniques were not used. Patients suffering from diarrhoea were used as the population of the studies and three studies were conducted on diarrhoeic patients with HIV. More than half of the studies (55.81%) were focusing on children less than equal to fifteen years of age. However, 39.53% of studies were considering all age groups (Fig 3 and Table 3).

### Prevalence of Gram-negative enteric bacterial pathogens

**Fig 4:**
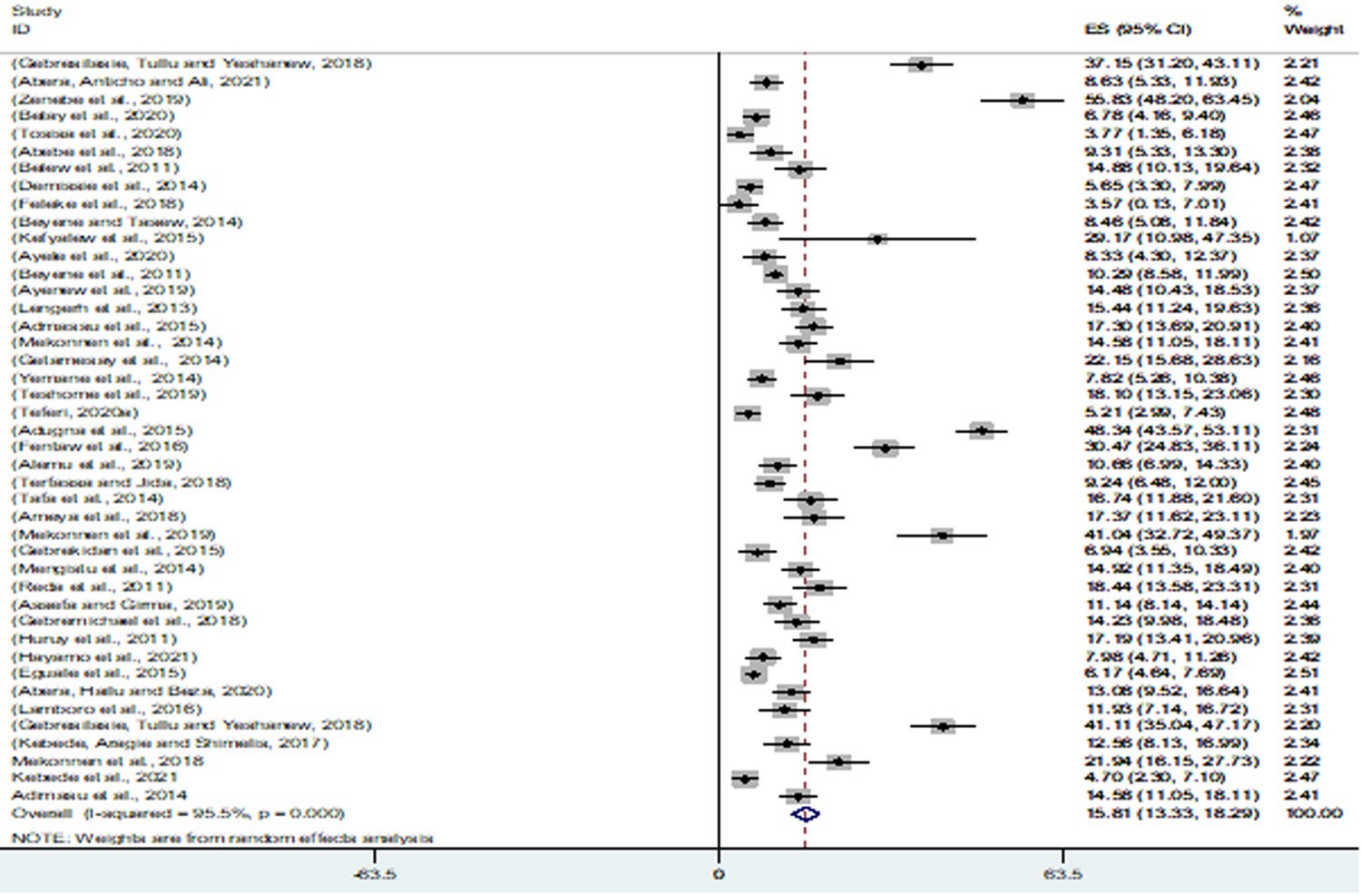
Forest plot of pooled prevalence estimates of Gram-negative enteric bacterial pathogens among diarrheic patients. The middle solid vertical line represents the minimum possible prevalence value (0). The dashed line represents the mean pooled prevalence estimate. The black diamond at the centre of the grey box represents the point prevalence estimate of each study and the horizontal line indicates the 95% confidence interval of the estimates. The grey box shows the weight of each study contributing to the pooled prevalence estimate. The last row represents the overall pooled prevalence estimate with a 95% confidence interval.

To isolate GNEBPs, in each study, 24 to 1,225 stool samples were collected. Totally, 13,350 stool samples were examined from 3,688 male and 3,822 female diarrheic patients and 1,962 (14.70%) samples were positive for GNEBPs. The minimum and maximum prevalence of GNEBPs in Ethiopia from diarrhoeic patients were 3.57% [27] and 55.83% [21]. The estimated pooled prevalence of GNEBPs in diarrheic patients from 43 studies was 15.81% (95% CI = 13.33–18.29) (Fig 4).

**Fig 5:**
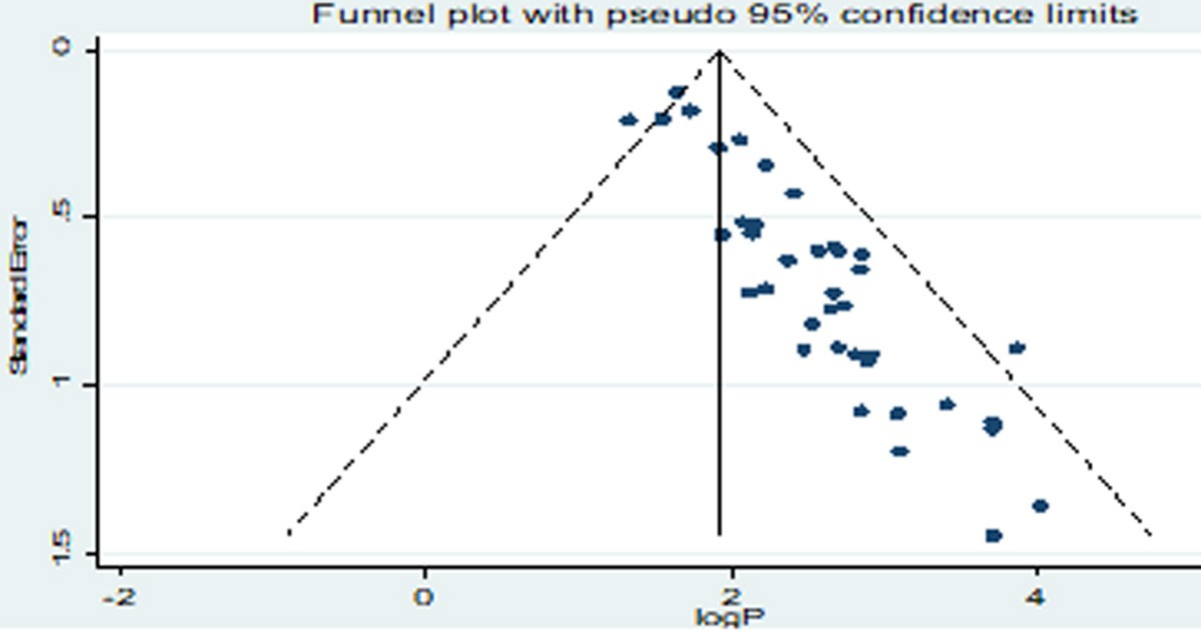
Funnel plot for the prevalence of Gram-negative enteric bacterial pathogens among diarrheic patients.

The distribution of the studies using a funnel plot (Fig 5) showed the asymmetrical distribution of effect estimates; hence, there was a publication bias. To minimize the effect of the bias, subgroup analysis was used. Regionally, the pooled prevalence of GNEBPs from diarrheic patients in Addis Ababa, Amhara, SNNPs and Oromia were 20.08, 16.67, 12.12, and 11.61%, respectively. The pooled prevalence based on the study period was 14. 91, 18.03 and 13.46% among studies from 2006–2010, 2011–2015 and 2016–2021, respectively. The pooled prevalence was the highest (20.35%) in children having age less or equal to 15 years, followed by all age groups (10.83%) and adults greater than 15 years (8.51%) (Table 2).

**Table 2:** The pooled prevalence of Gram-negative enterobacterial pathogens from diarrheic patients based on different subgrouping criteria.

Subgrouping criteria	Categories	No of studies	Sample examined	No Positive	Pooled prevalence ((%), CI)	I^2^% (p-value)
Regions	Addis Ababa	7	3532	517	20.08 (12.85–27.31)	97.9 (0.00)
	Amhara	13	4151	686	16.67 (10.08–22.48)	97.5 (0.00)
	SNNP	8	1847	227	12.12 (9.15–15.08)	75.7 (0.00)
	Oromia	8	2002	225	11.61 (7.98–15.24)	85.6 (0.00)
	Tigray	2	476	52	10.47 (3.33–17.61)	85.5 (0.00)
	Harari	3	1012	157	15.39 (13.17–17.67)	0.00 (0.38)
	Dire Dawa	1	196	43	21.94 (16.15–27.73)	-
	Gambella	1	134	55	41.04 (32.72–49.37)	-
Study period	2006–2010	4	2068	269	14.91 (10.44–19.39)	84.3 (0.00)
	2011–2015	21	6548	1106	18.03 (13.70–22.36)	96.8 (0.00)
	2016–2021	18	4738	587	13.46 (10.11–16.80)	93.8 (0.00)
Age of the study subjects	Children <15 years	24	7017	1308	20.35 (19.92–24.77)	96.8 (0.00)
	Adult > 15 years	2	458	39	8.51 (5.96–11.07)	0.00 (0.91)
	All age groups	17	5882	615	10.83 (8.69–12.98)	88.0 (0.00)

No = number, % = percent, SNNP = Southern Nations, Nationalities, and Peoples’ Region, CI = confident interval, I = Inconsistency Index.

Table 3 shows the types of bacterial isolates reported by published articles from diarrheic patients. *Shigella* spp. were the most frequent isolate (41.67%), followed by *S*. *enterica* (38.09%). The pooled estimate of *E*. *coli* was the highest (19.79%) among enteric bacterial isolates. The pooled prevalence of *Campylobacter* spp., *Shigella* spp. and *Salmonella enterica*. were 10.76, 6.24 and 5.06%, respectively.

**Table 3:** Bacterial isolates reported by published articles from diarrheic patient.

Bacterial isolates	No of Studies	Studies reporting the agent (%)	No of sample	No positive	Positives (%)	Prevalence (%)		
						Minimum	Maximum	Pooled (95% CI)
*Shigella* spp.	35	41.67	10,026	632	5.61	1.03	37.50	6.24 (4.66–7.81)
*Salmonella enterica*	35	38.09	11,360	644	5.67	0.38	62.50	5.06 (4.04–6.09)
*Escherichia coli*	9	7.1	2392	514	21.49	0.93	51.65	19.79 (10.48–29.09)
*Campylobacter* spp.	5	6.0	1079	123	11.40	4.12	16.74	10.76 (5.62–15.91)
*Citrobacter* spp.	1	1.2	253	10	3.95	-	-	-
*Enterobacter* spp.	1	1.2	163	2	1.23	-	-	-
*Klebsiella* spp.	1	1.2	163	11	6.75	-	-	-
*Proteus* spp.	1	1.2	163	7	4.29	-	-	-
Total	88*							

*An article may report one or more bacterial species from diarrheic patients; No = Number; % = Percent; CI = confident interval.

*Escherichia coli* was the most common (15.95%) isolate among children less than or equal to fifteen years of age whereas *Salmonella enterica* and *Shigella* spp. were common among studies that were focused on all age groups (Tables 1 and 4).

**Table 4:** Type bacterial isolates among different age groups.

Age category (years)	Type of bacterial Isolates	No of studies	No of Sample	No Positive	Pooled prevalence ((95% CI)
< 15	*Campylobacter* spp.	3	670	102	
	*E*. *coli*	8	2177	524	15.95 (14.52–17.38)
	*Salmonella enterica*	19	5893	296	2.95 (2.52–3.37)
	*Shigella* spp.	20	5767	344	3.95 (3.45–4.44)
Adult >15	*Campylobacter* spp.	1	180	8	
	*Salmonella* spp.	2	458	20	
	*Shigella* spp.	2	458	11	
All age groups	*Campylobacter* spp.	1	215	13	
	*E*. *coli*	1	215	2	
	*Salmonella enterica*	14	5067	381	3.21 (2.74–3.69)
	*Shigella* spp.	13	3859	221	3.14 (2.59–3.68)

No = Number; % = Percent; CI = confident interval.

### Drug resistance patterns of Gram-negative enteric bacterial pathogens

#### Drug resistance in *Shigella* spp

Twenty-six antimicrobial panels were used to assess the drug resistance pattern of *Shigella* spp. The highest percentage of resistance was reported among members of the penicillin group such as ampicillin (85.01%) and amoxicillin (82.07%). Resistance against the tetracycline group was also very common (67.01%). A considerable proportion of resistance was also reported among cephalosporin groups, particularly on the first-generation agents and erythromycin. Resistance was not reported against carbapenems (Table 5).

**Table 5:** Prevalence of *Shigella* spp. resistance to different antimicrobial agents in Ethiopia.

Antimicrobial Agents	The Main group of antimicrobial agents	No of studies	The total no of isolates tested	No of resistant isolate	Resistant isolate (%)	Pooled prevalence (%) (95% CI)	I^2^% (p-value)
Ampicillin	Penicillins	30	519	452	87.09	85.01 (79.79–90.22)	61.4 (0.00)
Amoxicillin	Penicillins	14	175	153	87.43	82.07 (74.05–89.65)	0.00 (0.47)
Augmentin (Amoxicillin and clavulanate potassium)	Penicillins and β-lactamase inhibitors	10	196	91	46.43	43.87 (22.5–65.24)	93.1 (0.00)
Tetracycline	Tetracyclines	22	404	309	76.49	67.01 (55.83–78.36)	89.9(0.00)
Doxycycline	Tetracyclines	2	20	16	80.00	-	-
Cephalothin	1^st^ G cephalosporins	4	57	40	70.18	70.18 (60.56–100.65)	100(-)
Cefoxitin	2^nd^ G cephalosporins	1	20	6	30.00	-	-
cefuroxime	2^nd^ G cephalosporins	1	20	13	65.00	-	-
Cefaclor	2^nd^ G cephalosporins	1	17	11	64.71	-	-
Ceftriaxone	3^rd^ G cephalosporins	14	151	16	10.60	29.53 (7.80–51.25)	79.5 (0.001)
ceftazidime	3^rd^ G cephalosporins	6	62	7	11.29	8.24 (0.42–16.05)	0.00 (0.46)
cefotaxime	3^rd^ G cephalosporins	2	20	4	20.00	-	-
Ceftizoxime	3^rd^ G cephalosporins	1	40	11	27.5	-	-
Chloramphenicol	Chloramphenicol	30	508	220	43.31	36.95 (29.65–44.24)	66.7 (0.00)
Kanamycin	Aminoglycosides	2	34	7	20.59	-	
Gentamicin	Aminoglycosides	27	443	88	19. 86	25.38 (17.78–32.99)	70.4
Amikacin	Aminoglycosides	3	22	2	9.09	14.29 (-4.04–32.62)	100(-)
Streptomycin	Aminoglycosides	1	32	31	96.87	-	-
Nalidixic acid	Fluoroquinolones	17	191	32	16.75	17.14 (11.34–22.94)	0.00(0.69)
Norfloxacin	Fluoroquinolones	14	229	14	6.11	8.34 (4.02–12.66)	0.00 (0.976)
Ciprofloxacin	Fluoroquinolones	28	468	32	6.84	11.86 (5.31–18.42)	65.6 (0.001)
Trimethoprim-sulphamethoxazole	Folic acid metabolism inhibitors	28	476	270	56.72	53.00 (44.34–61.67)	72.90 (0.00)
Meropenem	Carbapenems	2	11	0	0	-	-
Erythromycin	Macrolides	5	38	26	68.42	69.67(46.56–92.77)	59.9 (0.058)
Azithromycin	Macrolides	2	31	10	32.26	-	-
Clindamycin	Macrolides	1	8	4	50	-	-

No = Number; % = Percent; CI = confident interval, I = Inconsistency Index.

#### Drug resistance of *Salmonella enterica*

Twenty-five antimicrobial panels were used to assess the drug resistance patterns of *S*. *enterica*. The highest percentage of resistance was reported among members of the penicillin group such as ampicillin (64.98%) and amoxicillin (82.89%). Resistance against tetracycline and trimethoprim-sulphamethoxazole were also common. A considerable proportion of resistance was also reported against erythromycin and cephalosporin groups. Resistance was not reported against doxycycline, meropenem and azithromycin (Table 6).

**Table 6:** The pooled prevalence of *Salmonella enterica* resistance to different antimicrobial agents in Ethiopia.

Antimicrobial Agents	The main group of antimicrobial agents	No of studies	The Total no of isolates tested	No of resistant isolate	Resistant isolate (%)	Pooled prevalence (%) (95% CI)	I^2^% (p-value)
Ampicillin	Penicillins	29	588	447	76.02	64.98 (45.2–84.76)	96.5 (0.00)
Amoxicillin	Penicillins	12	255	223	87.45	82.89 (70.58–95.21)	62.0 (0.072)
Augmentin (Amoxicillin and clavulanate potassium)	Penicillins and β-lactamase inhibitors	8	185	75	40.54	45.34 (19.11–71.56)	95.2 (0.00)
Tetracycline	Tetracycline	24	555	287	51.71	54.59 (41.28–67.90)	90.03 (0.00)
Doxycycline	Tetracycline	2	34	0	0.00	-	-
Cephalothin	1st G cephalosporins	2	9	1	11.11	-	-
Cefaclor	2^nd^ G cephalosporins	1	4	4	100.00	-	-
Cefoxitin	2^nd^ G cephalosporins	1	33	9	27.27	-	-
Ceftizoxime	2^nd^ G cephalosporins	1	21	5	23.81	-	-
cefuroxime	2^nd^ G cephalosporins	2	55	16	29.09	-	-
Ceftriaxone	3^rd^ G cephalosporins	16	384	109	28.39	33.1 (7.88–58.33)	98.0 (0.00)
ceftazidime	3^rd^ G cephalosporins	5	27	4	14.81	29.22 (22.42–8086)	81.7 (0.02)
cefotaxime	3^rd^ G cephalosporins	2	7	2	28.57	-	-
Chloramphenicol	Chloramphenicol	29	629	260	41.34	42.39 (27.39–57.38)	96.1(0.00)
Kanamycin	Aminoglycosides	3	73	24	32.88	33.7 (22.72–44.69)	0.00 (0.67)
Gentamicin	Aminoglycosides	24	491	121	24.64	17.36 (7.57–27.15)	93.4 (0.00)
Amikacin	Aminoglycosides	3	26	1	3.85	-	-
Nalidixic acid	Fluoroquinolones	19	462	66	14.29	14.60 (9.23–19.97)	64.2 (0.00)
Norfloxacin	Fluoroquinolones	10	200	9	4.50	5.17 (1.79–8.55)	0.00(0.98)
Ciprofloxacin	Fluoroquinolones	28	28	28	5.29	7.66 (2.67–12.66)	74.8 (0.00)
Trimethoprim-sulphamethoxazole	Folic acid metabolism inhibitors	25	464	218	46.98	46.72 (29.74–61.69)	94.4 (0.00)
Meropenem	Carbapenems	2	20	0	0.00	-	-
Erythromycin	Macrolides	5	45	25	55.56	52.97 (37.96–68.72)	5.8 (0.364)
Azithromycin	Macrolides	1	5	0	0.00	-	-
Clindamycin	Macrolides	1	113	1	0.88	-	-

No = Number; % = Percent; CI = confident interval, I = Inconsistency Index.

#### Drug resistance of *Escherichia coli*

Sixteen antimicrobial panels were used to assess the drug resistance pattern of *E*. *coli*. The highest percentage of resistance was reported on agents like ampicillin (77.97%). Resistance against tetracycline (76.87%) and trimethoprim-sulphamethoxazole (66.97%) were also common. A considerable proportion of resistance was also reported among cephalosporin groups. Resistance was not reported against meropenem (Table 7).

**Table 7:** The pooled prevalence of *Escherichia coli* resistance to different antimicrobial agents in Ethiopia.

Antimicrobial Agents	The main group of antimicrobial agents	No of studies	The total no of isolates tested	No of resistant isolate	Resistant isolate (%)	Pooled prevalence (%) (95% CI)	I^2^ (p-value)
Ampicillin	Penicillins	6	446	359	80.49	77.97 (70.17–85.76)	71.4 (0.00)
Amoxicillin	Penicillins	1	47	5	10.64	-	-
Augmentin (Amoxicillin and clavulanate potassium)	Penicillins and B-lactamase inhibitors	5	339	204	60.18	64.78 (42.00–87.57)	95.0 (0.00)
Tetracycline	Tetracyclines	3	253	194	76.68	76.87 (71.69–82.05)	0.00 (0.563)
Ceftriaxone	3^rd^ G cephalosporins	5	284	11	3.87	2.91 (0.74–5.08)	11.5 (0.34)
Cephalothin	1st G cephalosporins	1	47	8	17.02	-	-
cefotaxime	3^rd^ G cephalosporins	1	204	50	24.51	-	-
Gentamicin	Aminoglycosides	6	388	102	26.29	19.72 (7.41–32.03)	88.03 (0.00)
Amikacin	Aminoglycosides	1	113	2	1.77	-	-
Chloramphenicol	Chloramphenicol	5	375	121	32.27	30.39 (20.95–39.84)	67.0 (0.016)
Nalidixic acid	Fluoroquinolones	5	224	38	16.96	16.71 (11.81–21.6)	0.00 (0.713)
Norfloxacin	Fluoroquinolones	2	206	20	9.71	-	-
Ciprofloxacin	Fluoroquinolones	7	439	27	6.15	5.57 (3.42–7.71)	0.00 (0.063)
Trimethoprim-sulphamethoxazole	Folic acid metabolism inhibitors	7	428	301	70.33	66.97 (56.21–77.71)	80.7 (0.00)
Meropenem	Carbapenems	1	133	0	0	-	-
Erythromycin	Macrolides	1	2	1	50	-	-

No = Number; % = Percent; CI = confident interval; I = Inconsistency Index.

#### Drug resistance of *Campylobacter* spp

Eighteen antimicrobial panels were used to assess the drug resistance patterns of *Campylobacter* spp. The highest percentage of resistance was reported for Cephalothin (81.52%). Resistance against ampicillin (65.61%) and trimethoprim-sulphamethoxazole (52.6%) were also common. Resistance was not reported against meropenem and azithromycin (Table 8).

**Table 8:** Pooled prevalence of *Campylobacter* spp. resistance to different antimicrobial agents in Ethiopia.

Antimicrobial Agents	The main group of antimicrobial agents	No of studies	The total no of isolates tested	No of resistant isolate	Resistant isolate (%)	Pooled prevalence (%) (95% CI)	I2 (p-value)
Ampicillin	Penicillins	4	110	72	65.45	65.61(44.90–86.32)	83.3 (0.000
Amoxicillin	Penicillins	1	20	16	80.00	-	-
Augmentin (Amoxicillin and clavulanate potassium)	Penicillins and B-lactamase inhibitors	1	44	16	36.36	-	-
Tetracycline	Tetracyclines	5	123	60	48.78	42.17 (20.30–64.05)	85.4 (0.00)
Doxycycline	Tetracyclines	3	90	16	17.78	18.94 (10.5–27.38)	0.00 (0.379)
Ceftriaxone	3^rd^ G cephalosporins	4	85	15	17.65	39.43 (0.96–77.91)	79.0 (0.029)
ceftazidime	3^rd^ G cephalosporins	1	8	2	25.00	-	-
Cephalothin	1^st^ G cephalosporins	3	102	91	89.22	81.52 (63.78–99.27)	63.2 (0.09)
Gentamycin	Aminoglycosides	5	123	29	23.58	30.70 (8.04–53.36)	88.0 (0.00)
Chloramphenicol	Chloramphenicol	5	123	26	21.14	28.03 (10.33–45.85)	74.4 (0.00)
Nalidixic acid	Fluoroquinolones	4	115	13	11.30	10.45 (4.89–16.00)	0.00 (0.711)
Norfloxacin	Fluoroquinolones	3	95	10	10.53	12.02 (4.99–19.05)	0.00 (0.665)
Ciprofloxacin	Fluoroquinolones	5	123	19	15.45	13.9 (7.87–19.94)	0.00 (0.528)
Trimethoprim-sulphamethoxazole	Folic acid metabolism inhibitors	5	123	67	54.47	52.6 (33.76–71.43)	78.4 (0.00)
Meropenem	Carbapenems	1	8	0	0.00	-	-
Erythromycin	Macrolides	5	123	37	30.08	35.72 (18.34–35.10)	78.4 (0.001)
Azithromycin	Macrolides	1	8	0	0.00	-	-
Clindamycin	Macrolides	2	82	28	34.15	-	-

No = Number; % = Percent; CI = confident interval; I = Inconsistency Index.

#### Drug resistance of other enteric bacterial species

The number of published articles on other enteric bacterial species from diarrheic patients was very limited and impossible to summarize. However, one study conducted by Zenebe *et al*. [21] reported the presence of *Klebsiella* spp., *Proteus* spp., *Enterobacter* spp. in under-five children with diarrhoea. These bacteria were showing antimicrobial resistance character as indicated in Table 9.

**Table 9:** Pooled prevalence of other enteric bacterial resistance to different antimicrobial agents in Ethiopia.

References	bacterial isolate	Ampicillin			Chloramphenicol			Gentamicin			Nalidixic acid			Tetracycline			Ciprofloxacin			Trimethoprim-sulphamethoxazole			Ceftriaxone			Amoxicillin			Cephalothin		
		No isolate tested	No resistance	%	No isolate tested	No resistance	%	No isolate tested	No resistance	%	No isolate tested	No resistance	%	No isolate tested	No resistance	%	No isolate tested	No resistance	%	No isolate tested	No resistance	%	No isolate tested	No resistance	%	No isolate tested	No resistance	%	No isolate tested	No resistance	%
[21]	*Klebsiella*	11	3	27.27	11	2	18.18	11	4	36.36	11	1	9.09	11	9	81.82	11	0	0.00	11	8	72.73	11	0	0.00	11	4	36.36	11	2	18.18
[21]	*Proteus*	7	2	28.57	7	1	14.29	7	3	42.86	7	4	57.14	7	2	28.57	7	0	0.00	7	2	28.57	7	1	14.29	7	3	42.86	7	0	0.00
[21]	*Enterobacter*	2	1	50.0	2	0	0.00	2	1	50.0	2	1	50.0	2	0	0.0	2	0	0.0	2	0	0.0	2	0	0.0	2	0	0.0	2	0	0.00

### Multidrug resistance

Out of 43 published articles on enteric bacterial pathogens, 32 (74.42%) reported multidrug-resistant (MDR) characters among the isolates. Among 1470 bacterial isolates, 1104 (75.52%) with a pooled prevalence of 70.56% (CI = 64.56–76.77%) were resistant to three or more antimicrobial agents (multidrug resistance). The report showed that the pooled prevalence of MDR in *Campylobacter* spp., *Shigella* spp., *E*. *coli* and *S*. *enterica* were 80.78, 79.08, 78.20 and 59.46%, respectively (Table 10).

**Table 10:** Multidrug resistance pattern of enteric bacteria pathogens from diarrheic patients.

Type bacterial isolate	No of studies	The total no of isolates tested	No of multidrug-resistant isolates	Multidrug-resistant isolate (%)	Pooled prevalence (%) (95% CI)	I^2^ (p-value)
*Shigella* spp.	24	443	363	81.94	79.08 (72.19–85.97)	68.2 (0.00)
*Salmonella enterica*	23	496	317	63.91	59.46 (46.13–72.79)	91.1 (0.00)
*Escherichia coli*	6	410	332	80.98	78.20 (67.46–88.93)	84.7 (0.00)
*Campylobacter* spp.	4	110	87	79.09	80.78 (65.04–96.52)	94.6 (0.00)
*Klebsiella* spp.	1	11	5	45.45	-	-
		1470	1104	75.52		

No = number, % = percent; CI = confidence interval, I = Inconsistency Index.

## Discussion

Diarrhoea is a common health problem, causing mortality and morbidity for thousands of people around the globe. Both infectious and non-infectious agents can induce the problem, among the infectious agents, enteric bacterial pathogens like diarrheagenic *E*. *coli*, *S*. *enterica*, *Shigella* spp., and *Campylobacter* spp. play important roles in the induction or severity of diarrhoea [61]. Determining their burden in a given population is very essential to design strategies for the reduction of the incidence and influences of diarrhoea. The minimum and maximum prevalence of GNEBPs in Ethiopia from diarrhoeic patients were 3.57% [27] and 55.83% [21], respectively. The pooled prevalence of EBP isolates from the stool of diarrheic patients in Ethiopia was 15.81% (CI = 13.33–18.29). In line with this finding, Getie *et al*. [62] reported 13.2% prevalence of GNEBP in other groups of population (food handlers) in Gondar town, Northwest Ethiopia. On the other hand, Shah *et al*. [63] reported a 33.62% prevalence of GNEBP in Kenya. The difference may be due to the detection methods since they use molecular techniques in addition to the conventional culturing methods.

Almost all studies (97.67%) were institutional, and samples were collected from patients visiting health facilities. Focusing only on health facilities may not reflect the overall prevalence of GNEBPs and their drug resistance patterns in the country. Since some GNEBP infection cases may not arrive at health institutions and widespread use or misuse of antimicrobial drugs in the community may accelerate the occurrence of antimicrobial resistance [64,65].

Sub-grouping of the prevalence of GNEBPs based on the studies conducted in different regions of the country showed that the pooled prevalence was high in Addis Ababa (20.08%). In line with this, a spatial variation across the regions of Ethiopia was reported by Bogale *et al*. [66]. The difference based on the study period was not significant. However, a declining pattern of diarrhoea at the national level was reported by Bogale *et al*. [66]. Published articles were from 8 regions of Ethiopia, published articles were not found in Afar, Somali and Benishangul Gumuz regions of the country in this study. Hence, the pooled prevalence was calculated from 8 regions ignoring others. However, the scenario may not be equivalent to the region of the country having no reports.

Grouping of study participants based on their age showed that the pooled prevalence was the highest (20.35%) in children having age below or equal to 15 years which indicated that children are more exposed to the GNEBPs and express severe syndromes to visit health facilities. In line with this, Kotloff [4] and Havelaar *et al*. [67] reported that children contribute a huge proportion of diarrheal diseases in the world.

Diarrheagenic *E*. *coli*, *Shigella* spp., *S*. *enterica* and *Campylobacter* spp. were the most common isolates among GNEBPs. In line with this, Getie *et al*. [62] reported that *Shigella* spp. enterohemorrhagic *E*. *coli* (EHEC) and *S*. *enterica* were important isolates of GNEBPs among food handlers. In this study, the pooled estimate of *E*. *coli* was the highest (19.79%) among enteric bacterial pathogens. The result is very close to the report of Zenebe *et al*. [68] who reported that the pooled prevalence of *E*. *coli* was 25% in Ethiopia. A 33.8% pooled prevalence was reported by Oppong *et al*. [69] in Sub-Sharan Africa. Oppong *et al*. [69] also found that *E*. *coli* detection was the highest in the East African region and lowest in the middle part of Africa. The difference may be due to the number of studies in the summary and the targeted strain of *E*. *coli*. For example, in a single study in Niger, 11.1% of diarrhoeic children were positive for diarrheagenic *E*. *coli* [70]. Similarly, the Global enteric multicentre study on infants and children showed that diarrheagenic *E*. *coli* was among the four major pathogens responsible for diarrhoea in low-income and middle-income countries [71].

The pooled prevalence of *Campylobacter* spp. in this analysis was 10.76%. In line with this report, Kassie *et al*. [72] reported a prevalence of 10.5% among children in Denbia district, Ethiopia and 13.8% prevalence was also reported by Gedlu and Aseffa [73] among children in northwest Ethiopia. Oppong *et al*. [69] reported a 12.3% pooled prevalence of *Campylobacter* in East Africa. A high rate of *Campylobacter* infection among children was also reported in Kenya [63]. In contrary to the report of this study, Fletcher *et al*. [74] reported a pooled prevalence of 2.7% in Sub-Saharan countries. The difference may be related to the area coverage, disease prevention and control practices.

The pooled prevalence of *Shigella* spp. in this study was 6.24% which is equivalent to the report of Hussen *et al*. [75]. According to their report, the pooled prevalence of *Shigella* spp. in Ethiopia was 6.6%. Similarly, Oppong *et al*. [69] reported a pooled prevalence of 5.6% from children under five and Fletcher *et al*. [74] 4.3% of children aged less than 12 years in Sub-Saharan countries.

The pooled prevalence of *S*. *enterica* was 5.06% which is almost comparable with the reported pooled prevalence by Abate and Assefa [76], which was 4.8% among human stools and animal origin foods in Ethiopia.

Resistance to antimicrobial agents is a natural evolutionary process for the bacteria, however, the process is accelerated by human activities in terms of antimicrobial usage patterns and infection control or prevention practices. The risks are very high in developing countries like Ethiopia where there is a widespread use or misuse of antimicrobial agents with a high burden of infectious diseases [76–79].

In this meta-analysis, *Shigella* isolates were more resistant to the penicillin group of antimicrobial agents like ampicillin (85.01%) and amoxicillin (82.07%). A high percentage of resistance against ampicillin (83.1%) and amoxicillin (84.1%) were also reported by Hussen *et al*. [75]. There were also reports of drug resistance among the first-line drugs like ciprofloxacin (11.86%) and ceftriaxone (29.53%) for the treatment of Shigellosis. Resistance development against such types of antimicrobial agents was also reported by Hussen *et al*. [75]. They reported 8.9 and 9.3% resistance against ciprofloxacin and ceftriaxone, respectively.

In this analysis, high proportions of *Salmonella* isolates were also resistant against the penicillin group of antimicrobial agents like ampicillin (64.98%) and amoxicillin (82.89%). In line with this Tadesse [80] was also reported a high percentage (86.01%) of resistance of *Salmonella* against ampicillin and/or amoxicillin. Resistance of *Salmonella* isolates to fluoroquinolone like ciprofloxacin (2.27%) and third-generation cephalosporin (ceftriaxone 16.68%) were also reported. Similarly, according to Tadesse’s report, the pooled prevalence of ciprofloxacin resistance among *Salmonella* isolates was 3.61% [80] and a prevalence of 2.9% of resistance against ciprofloxacin was also reported in Iran [81].

Resistance was common among *E*. *coli* isolates in this analysis, particularly on antimicrobial agents like ampicillin (77.97%), tetracycline (76.87%) and trimethoprim-sulphamethoxazole (66.97%). Resistance of *E*. *coli* against a wide array of antimicrobials was also reported by Pormohammad *et al*. [82], Zenebe *et al*. [68] and Tuem *et al*. [83]. Resistance in non-pathogenic strains of *E*. *coli* may not have a direct effect on health, however, non-pathogenic resistant strains may acquire virulence genes and induce disease that may not be treated easily or non-pathogenic strains having resistant character may act as a reserve for the resistant character for other bacteria [82].

Among *Campylobacter* isolates in this analysis, the highest percentage of resistance was reported on antimicrobial agents like cephalothin (81.52%), ampicillin (65.61%) and trimethoprim-sulphamethoxazole (52.60%). A resistance pattern of 92.3–100% to erythromycin and the β—lactams, 61.5–86.7% to trimethoprim-sulfamethoxazole, 92.3–93.3% to tetracycline, 46.2–80% to chloramphenicol, 0–60% to aminoglycosides and 0% to imipenem were reported among *Campylobacter* spp. in Ghana [84].

In this study, among 1470 bacterial isolates 1104 (75.52%) with a pooled prevalence of 70.56%, were resistant to three or more antimicrobial agents (multidrug resistance) (MDR). In line with this finding, Alemayehu [85] reported that the pooled prevalence of multidrug resistance was 70.5% among bacterial isolates in Ethiopia. Another, meta-analysis study on multidrug resistance by Abayneh *et al*. [86] reported the pooled prevalence of 80.5% among Gram-negative bacteria. It was also very close to the reports from India (66.12%) [87] and Egypt (65.5%) [88,89]. In contrast to our finding in Ethiopia, a lower prevalence of MDR has been reported from Germany (60%) [90], Nepal (42.6%) [91], Australia (36%) [92], Indonesia (28.7%) [93], the USA (27%) [94], Spain (34.5%) [95] and France (11.6%) [96]. Several factors may play a role in the difference including the magnitude and style of antimicrobial use and infection prevention practices.

The MDR report among *Campylobacter* isolates in this analysis (80.78%) was higher than the report from Bangladesh (28.8%) [97] and Kenya 50% [98] but lower than the report from Ghana (97%) [84]. This may be due to differences in the use of antimicrobial agents, the area coverage, sample type and technique of detections. The MDR report of *E*. *coli* in this analysis (78.20) was not in line with other reports like 50% prevalence in Nigeria [99], 40% in Spain [95], 22% among human isolates in the world [82], 26% in China [100], 39.8% in Egypt isolates from animals [101], 28% in low and middle-income countries [102]. The MDR report of this meta-analysis among *Shigella* isolates (79.08) was almost similar to the report by Hussen *et al*. [75] (83.2%) but it was lower than other reports from Iran (89.4%) [103], and Bangladesh (94%) [104]. However, it was higher than other reports such as 53.8% among migrants in Europe [74], 60% in Kenya [98] and 19% in Somalia [105]. Factors like the magnitude and style of antimicrobial use and infection prevention practices may play roles in the differences.

The development of MDR character among *S*. *enterica* is also an important public health concern around the globe [106]. In this analysis, the prevalence of MDR character among *Salmonella* isolate was 59.46% which is comparable with the MDR report of Garedew *et al*. [107] (46.2%) among food handlers in Gondar. However, it was lower than the report from Dagnew *et al*. [108] (76%) and Admassu *et al*. [109] (100%). The difference may be due to the target population and study methods (single versus pooled meta-analysis reports).

## Limitations of the study

Some of the studies included in the analysis were targeting the most common bacterial pathogens that did not rule out the absence of others. Therefore, for bacteria that are thought to be less frequent, the reported prevalence may not accurate. Publication bias and heterogenicity were observed in the analysis, but attempts were made to reduce their impact on the analysis by following the random effect model and subgrouping. However, these may not totally avoid their impact on the interpretation of the pooled results.

## Conclusion

According to this analysis, the burden of Gram-negative enteric bacterial pathogens (GNEBPs) was high and may be considered a major cause of diarrhoea in Ethiopia. A significant proportion of the isolates exhibited resistance to the commonly used antibacterial agents which are expected to affect the treatment response and cost, morbidity and progression of the infection. *Shigella* spp., *S*. *enterica*, *E*. *coli* and *Campylobacter* spp. were the commonly isolated GNEPB from diarrheic patients in the country. The pooled estimate of *Campylobacter* spp. was the highest followed by *E*. *coli*, *Shigella* spp. and *S*. *enterica*. Resistance to antimicrobial agents was most common among the penicillin groups, followed by tetracycline, and trimethoprim-sulphamethoxazole. However, there were also resistant strains against very relevant drugs for the treatment of GNEBP such as fluoroquinolones and thirdgeneration cephalosporins. Almost all isolates were susceptible to meropenem.

Since the incidences of these bacterial diseases are related to hygiene, all activities that enhance hygienic practices (clean water and food, handwashing, proper use of latrine) must be advocated and implemented. Performing drug sensitivity tests for suspected diarrheagenic bacteria is extremely advantageous to select the appropriate antimicrobial drugs for the treatment. The antimicrobial resistance surveillance system must be established to understand the trend of resistance among pathogenic bacteria and to plan and implement mitigating strategies like proper control and prevention of infectious diseases and antimicrobial stewardship programs. Almost all studies were using conventional techniques for confirmation of the isolates, thus, adding molecular methods in the future will increase analysis precision.

## Supporting information

S1 File Forest plot of pooled prevalence estimates of Gram-negative enteric bacterial pathogens in different regions of Ethiopia. (DOCX)

S2 File Forest plot of pooled prevalence estimates of Gram-negative enteric bacterial pathogens subgrouping based on the study period. (DOCX)

S3 File Forest plot of pooled prevalence estimates of Gram-negative enteric bacterial pathogens subgrouping-based age of the study subjects. (DOCX)

S4 File Frost plot of the studies on *Shigella* species. (DOCX)

S5 File Frost plot of the studies on *Salmonella enterica*. (DOCX)

S6 File Frost plot of the studies on *Escherichia coli* strains. (DOCX)

S7 File Frost plot of the studies on *Campylobacter* species. (DOCX)

S8 File Frost plot of the studies on multidrug resistance. (DOCX)

S9 File Frost plot of the studies on multidrug resistance of *Shigella* species. (DOCX)

S10 File Frost plot of the studies on multidrug resistance of *Salmonella enterica*. (DOCX)

S11 File Frost plot of the studies on multidrug resistance of *E*. *coli*. (DOCX)

S12 File Frost plot of the studies on multidrug resistance of *Campylobacter* species. (DOCX)

S13 File Frost plot of the studies on the prevalence of *Escherichia coli* in children less than or equal to 15 years of age. (DOCX)

S14 File Frost plot of the studies on the prevalence of *Escherichia coli* in children less than or equal to 15 years of age. (DOCX)

S15 File Frost plot of the studies on the prevalence of *Shigella* spp in children less than or equal to 15 years of age. (DOCX)

S16 File Frost plot of the studies on the prevalence of *Salmonella enterica* in all age groups. (DOCX)

S17 File Frost plot of the studies on the prevalence of *Shigella* species in all age groups. (DOCX)

## Abbreviations

GNEBPs: Gram-Negative Enteric Bacterial Pathogens
HIV: Human Immunodeficiency Virus
I^2^: Inconsistency Index
KAP: Knowledge, Attitude and Practices
MESH: Medical Subject headlines
PRISMA: Preferred Reporting Items for Systematic Reviews and Meta-Analyses
SNNP: South Nation Nationalist People’sregion
UN: United Nations
USA: United State of America
WHO: World Health Organization
