# Supplementary material for: Prevalence and drug resistance patterns of Gram-negative enteric bacterial pathogens from diarrheic patients in Ethiopia: A systematic review and meta-analysis
Source: PLoS One. 2022 Mar 16;17(3):e0265271. doi: 10.1371/journal.pone.0265271 (PMC8926281; doi:10.1371/journal.pone.0265271)
Supplement: S13 File — (DOCX) [file pone.0265271.s013.docx]

**Supplementary document 13**

**S13. Frost plot of the studies on the prevalence of *Escherichia coli* in children less than or equal to 15 years of age**

The middle solid vertical line represents the minimum possible prevalence value (0). The dashed line represents the mean pooled prevalence estimate. The black dot at the centre of the grey box represents the point prevalence estimate of each study and the horizontal line indicates the 95% confidence interval of the estimates. The grey box shows the weight of each study contributing to the pooled prevalence estimate. The last row represents the overall pooled prevalence estimate with a 95% confidence interval.
