# Supplementary material for: Prevalence and drug resistance patterns of Gram-negative enteric bacterial pathogens from diarrheic patients in Ethiopia: A systematic review and meta-analysis
Source: PLoS One. 2022 Mar 16;17(3):e0265271. doi: 10.1371/journal.pone.0265271 (PMC8926281; doi:10.1371/journal.pone.0265271)
Supplement: Attachment — Submitted filename: Response to Reviewers.docx [file pone.0265271.s018.docx]

**Comments and Feedback**

Prevalence and drug resistance patterns of Gram-negative enteric bacterial pathogens from diarrheic patients in Ethiopia: A systematic review and meta-analysis

|  | **Comments** | **Corrections/ Feedback** |
| --- | --- | --- |
|  | **Reviewer 1** |  |
| 1. | Published articles were not found in Afar, Somali, and Benishangul Gumuz regions of the country in this study. It is suggested to be analysed in the discussion section | Statements addressing this issue are included in the discussion section, line 328-331 |
| 2. | Table 1 lists the detection techniques and methods used in the article should be better. | The issue is addressed in revised Table 1; lines 176 – 186. |
| 3. | Analysis of Common Pathogens by Adults and Children in table 3 maybe useful | A new table (Table 4) is added in the document showing the type of bacterial isolates among different age groups |
| 4. | From table 5 to table 7, If there are too few isolates for statistical analysis, it is recommended to delete relevant information to avoid bias in drug resistance | The comment has been addressed. The tests were conducted on isolates less than 10 or if the number of studies are too few, we didn’t report the pooled prevalence. |
| 5. | Analysis of Multi-drug resistance pattern by Adults and Children in table 9 may be useful. | Yes, the proposed analysis may be useful, however, we didn’t find such information in the selected articles. |
|  | **Review 2** |  |
| 1 | The study primarily focuses on *E. coli, Campylobacter, Shigella*, and *Salmonella*. While all except *E. coli* are known to be pathogenic, there is no description on how the studies differentiated DEC from commensal *E. coli*. The authors have touched upon the fact that resistance of commensal *E. coli* may not be relevant but may affect those of DEC but methods employed by the various reports to identify DEC is completely missing. In particular, it is said that the studies used conventional methods and not molecular methods and therefore it leaves us wondering how they were differentiated. | Generally, bacteria were isolated, characterized and confirmed by culturing and conventional biochemical tests, and antibiotic resistance was measured by disk diffusion. However, molecular techniques were not used (line 166). The articles didn’t focus on detection and confirmation methods of individual DEC. Most articles have considered *E. coli* as a whole (both pathogenic or non-pathogenic) but only a few tried to confirm *E. coli* O157: H7 strain only by using Sorbitol-MacConkey media (For example, Ayenew *et al.,* 2019).  The relevance of determining the antimicrobial resistance pattern of the non-pathogenic strain of *E. coli* has been explained in the document (lines 389-391) |
| 2 | Was there any information available on co-infections? | Yes, there were co-infection in a patient, which is already indicated in table 3. Total reported bacterial species were 88 from 43 articles. |
| 3 | We often see that GNEBPs constitute only a minor portion of childhood diarrheal. For e.g. Rotavirus is responsible for almost 30-40% of diarrhoeas in developing countries. In one particular study in India, viral infections accounted for almost 80% of all cases of diarrhoea. Therefore, the rampant use/misuse of antimicrobials in developing countries in the first place uncalled for, resulting in unnecessary increase in AMR. The authors should try to bring out this fact also in the discussion section /limitation section so as to advocate rational use of antibiotics. | Diarrhoea is a multi-factorial problem, that can be caused by both infectious and non-infectious agents. The infectious agents include viruses, bacteria and protozoan parasites. Which one will dominate may vary among locations, seasons, age, sanitary status and other factors. Viruses may be the dominant ones based on these factors. However, the role of bacteria may be high in other places. Some examples   1. In China, Shen *et al.,* [1], identified 12 gastrointestinal pathogens of acute infectious diarrhoea and they found that bacteria were the dominant causes. 2. WHO [2] stated that rotavirus and Escherichia coli are the two most common etiological agents of moderate-to-severe diarrhoea in low-income countries 3. Kotloff [3] studied the burden and aetiology of diarrheal illness in developing countries and found four pathogens (rotavirus, *Cryptosporidium*, *Shigella,* and ETEC) were responsible for most diarrheal illnesses. 4. Kotlof et al. [4] also emphasized some of the bacterial species as contributing factors for the induction of diarrhoea.   Therefore, there is ample evidence that shows the significant role of bacteria, especially GNEBPs, in the induction of diarrhoea. Even though viruses like rotavirus contribute a large portion for the induction of diarrhoea, they were not the focus of this study.  Advocating rational use of antibiotics is already emphasized in the document (lines 368, 407 and other lines in the discussion, in line 453, establishing antimicrobial stewardship program has been recommended to optimise the use of antimicrobial agents), since it is highly related to AMR. |
| 4 | In view of the fact that studies with certain antibiotics taken in the panel e.g Meropenem (Carbapenams), 2nd and 3rd generation Cephalosporins etc are extremely low (1 or 2 only) sweeping statements on not finding resistance to these antibiotics may not be appropriate (only a few of the total isolates were actually tested against these antibiotics). | The comment has been addressed. For tests conducted on isolates less than 10 or if the number of studies is too few, we didn’t report the pooled prevalence. |
| 5 | It would have been better to provide trends in antimicrobial resistances in various pathogens that would help clinicians for their choice of empirical treatment, when absolutely required. | This issue is addressed in the document in lines 243-300; the resistance pattern of individual agents was assessed and described. |
| 6 | There are other typos and small grammatical errors e.g many words have got joined together (might be due to PDF conversion or ununiform versions of MSO). There is too much use of the word 'causes' in Introduction lines 68-72; Line 164 after Figure 2, Almost all studies (97.67%) ere institutional-based... may be replaced with 'institution-based'; Line 233 'Resistant against...' may be replaced with 'Resistance against...'. | All typos and grammatical errors were corrected in the revised document. |

**References**

[1]	H. Shen *et al.*, “The 12 Gastrointestinal Pathogens Spectrum of Acute Infectious Diarrhea in a Sentinel Hospital, Shenzen, China.,” *Front. Microbiol.*, vol. 7, pp. 1-9. doi: 10.3389/fmicb.2016.01926., 2016.

[2]	WHO, “Diarrhoeal disease,” 2017. https://www.who.int/news-room/fact-sheets/detail/d.

[3]	K. L. Kotloff, “The Burden and Etiology of Diarrheal Illness in Developing Countries,” *Pediatr. Clin. North Am.*, vol. 64, pp. 799–814, 2017.

[4]	K. L. Kotloff *et al.*, “The incidence , aetiology, and adverse clinical consequences of less severe diarrhoeal episodes among infants and children residing in low-income and middle-income countries: a 12-month case-control study as a follow-on to the Global Enteric Multicenter S,” *Lancet Glob. Heal.*, vol. 7, no. 5, pp. e568–e584, 2019.
